# Parental Internalizing Psychopathology and PTSD in Offspring after the 2012 Earthquake in Italy

**DOI:** 10.3390/children8100930

**Published:** 2021-10-17

**Authors:** Barbara Forresi, Marcella Caputi, Simona Scaini, Ernesto Caffo, Gabriella Aggazzotti, Elena Righi

**Affiliations:** 1 Department of Psychology, Sigmund Freud University (Milan), Ripa di Porta Ticinese, 77-20143 Milan, Italy; s.scaini@milano-sfu.it; 2 Department of Life Sciences, University of Trieste, Via E. Weiss, 2-34128 Trieste, Italy; marcella.caputi@units.it; 3 Department of Biomedical, Metabolic, and Neural Sciences, University of Modena and Reggio Emilia, 287-41125 Via Campi, Italy; ernesto.caffo@unimore.it (E.C.); gabriella.aggazzotti@unimore.it (G.A.); elena.righi@unimore.it (E.R.)

**Keywords:** earthquake, trauma, PTSD, parental psychopathology, internalizing disorders, youths, children, adolescents

## Abstract

Post-traumatic stress disorder (PTSD) is common in youths after earthquakes, with parental psychopathology among the most significant predictors. This study investigated the contribution and the interactional effects of parental internalizing psychopathology, the severity of exposure to the earthquake, and past traumatic events to predict PTSD in offspring, also testing the reverse pattern. Two years after the 2012 earthquake in Italy, 843 children and adolescents (9–15 years) living in two differently affected areas were administered a questionnaire on traumatic exposure and the UCLA PTSD Reaction Index. Anxiety, depression, and somatization were assessed in 1162 parents through the SCL-90-R. General linear model showed that, for offspring in the high-impact area, predictors of PTSD were earthquake exposure, past trauma, and parental internalizing symptoms, taken individually. An interaction between earthquake exposure and parental depression or anxiety (not somatization) was also found. In the low-impact area, youth PTSD was only predicted by earthquake exposure. The reverse pattern was significant, with parental psychopathology explained by offspring PTSD. Overall, findings support the association between parental and offspring psychopathology after natural disasters, emphasizing the importance of environmental factors in this relationship. Although further research is needed, these results should be carefully considered when developing mental health interventions.

## 1. Introduction

As the frequency of large-scale disasters and emergencies is increasing around the globe, children and adolescents are among the most vulnerable populations for the development of negative mental health consequences. Several studies confirmed that youths exposed to disasters show an increased prevalence of psychological disorders such as post-traumatic stress disorder (PTSD), depression, anxiety/somatization disorders, substance abuse, and sleep problems [1,2,3,4,5,6]. One of the most common sequelae is represented by PTSD [3,7,8,9,10,11,12,13,14], with a prevalence in youths ranging between 5% and 60% [15]. High rates of PTSD have been detected among children and adolescents not only in the aftermath but also several years after earthquakes [7,10,11,16,17,18,19,20,21,22,23,24,25,26,27]. According to a recent meta-analysis of studies conducted among children and adolescents exposed to earthquakes and floods [28], the pooled prevalence of PTSD was 19.2%, 30%, 24.4%, and 20.4% in the first, second, third, and fourth six-month intervals after the event.

Considering the long-lasting effects of PTSD and its negative impact across development, identification of its predictors is of primary importance. Several pre-traumatic and peritraumatic predictors of PTSD in children and adolescents after disasters have been identified [15,28,29,30], such as demographic characteristics (e.g., being female), pre-disaster events and functioning (e.g., previous traumatic events and psychological difficulties), and severity of disaster exposure (e.g., being trapped, experiencing injuries and losses). While prior trauma was identified as a small to medium risk factor, the severity of exposure appeared as the strongest risk factor [29]. According to the meta-analytic examination by Furr and colleagues [30], for example, higher death toll, proximity, and personal loss were each associated with increased post-traumatic stress symptoms (PTSS) in youths.

Among post-traumatic factors, the meta-analysis by Trickey and colleagues [29] highlighted the important role played by parental psychological problems and family functioning in youth PTSD. This association between psychological difficulties in parents and offspring can be referred not only to a genetic predisposition and therefore to common biological bases for emotional processing but also to difficult parenting or other family challenges [31,32]. Parents represent the primary support for children and adolescents after traumatic events and disasters [33], and their psychopathology may have a cascade effect on several other variables such as parenting styles [34], parent-youth communication, and relationships [35]. Moreover, according to the model of Pynoos and colleagues [36], parents can moderate the impact of the event, of proximal reminders and secondary stressors, supporting the cognitive appraisal of what occurred, as well as the ongoing adjustment. It is therefore evident that youth mental health after traumatic events and disasters can be best understood only considering their family context, according to a socio-ecological model (e.g., the work of [31,37,38,39,40,41]).

The relevance of parental psychopathology as a risk factor for PTSD in offspring has been extensively studied after several potentially traumatic events, ranging from hospitalization and pediatric burn [42,43,44,45,46,47,48,49] to traffic injuries, community violence, industrial disasters, war, and terrorist attacks [32,50,51,52,53,54,55,56,57,58]. The association has also been detected after earthquakes [12,16,59,60,61,62,63,64] and other natural disasters [41,65,66,67,68]. However, existing research is still scant and affected by many methodological flaws, including poor in-depth analysis of the role of parental distress on child outcomes, a limited consideration of bidirectional associations, the use of a single source of information (parent or child) [69,70].

In their cross-sectional study, Spell and colleagues [41] found that maternal distress predicted PTSS in children exposed to Hurricane Katrina. Instead, Kiliç and colleagues [62,63] found that six months after the Bolu earthquake in Turkey, PTSD in children was affected by their father’s traumatic stress and depression. Similarly, in their survey of survivors from three disasters, North and colleagues [69] found that parental psychopathology was associated with child emotional and behavioral problems. A recent meta-analysis [31] confirmed that parental PTSD is associated with children’s psychological distress.

The same topic was explored in a longitudinal perspective [60,71]. After the Great East Japan Earthquake, Honda and colleagues [60] investigated the impact of parental PTSD on children’s mental health using multivariate logistic regression analysis. While cross-sectionally no significant association was found, parental PTSD symptoms at baseline were a significant predictor of children’s internalizing problems measured three years later. In another study, children exposed to Hurricane Katrina whose mothers had a chronic PTSD trajectory also presented severe post-traumatic stress symptoms two years later [71].

A smaller number of studies focused on the opposite relationship, investigating whether children’s PTSD has an impact on parental mental health. In a recent article, Pfefferbaum and North [72] explored cross-sectionally the influence of children’s psychopathology on their parents’ mental health in a large sample including 1066 children and 556 parents exposed to different disasters and terrorist attacks, with findings supporting the direction from child psychopathology to parental PTSD.

Similarly, in their longitudinal study, Shi and colleagues [64] studied 688 parent-adolescent dyads after the 2008 Wenchuan earthquake, finding that parental PTSS at 1 year prospectively predicted teens’ PTSS after 18 months; on the other side, youth PTSS at 1 year predicted maternal but not paternal PTSS 18 months later. This study indeed highlights a causal role of parental PTSS on PTSS in offspring and a bidirectional pattern of influence between maternal and youth psychopathology.

Inconsistent conclusions were drawn by Juth and colleagues [61]. Using the interesting Actor-Partner Interdependence Model (APIM) after a major earthquake in Indonesia, researchers observed cross-sectionally that parental post-traumatic symptoms resulted in being associated with children’s distress: the reverse was not true, suggesting the existence of a unidirectional path of influence.

In conclusion, notwithstanding a strong consensus on the relationship between parental psychopathology and youth PTSD after disasters, research in this field is still scant and mostly focused on parental PTSD [41,60,65,67,73], parental depression [62,63,67], or general psychological distress [41]. To our knowledge, no study focused on a broader pattern of parental internalizing psychopathology, including somatization, anxiety, and depression.

Furthermore, while the relationship between parental psychopathology and offspring PTSD found significant support, a reciprocal pattern of influence has been suggested with preliminary evidence [64,72], without clear conclusions. Notably, none of the previous studies looked at parental psychopathology in interaction with the severity of exposure to the earthquake and past trauma to determine PTSD in offspring.

The present cross-sectional study aimed at contributing to the literature on natural disasters in the light of the important role played by parental mental health in offspring PTSD [74,75,76].

The overall purpose was to examine the relationship between parental internalizing psychopathology and PTSD in offspring two years after the earthquake that hit Northern Italy in 2012, comparing students living in the area most affected by the earthquake with others living in a nearby control area. As to our knowledge, no previous study was able to compare the psychopathology of parents and their offspring in two differently exposed samples.

In the first place, we were interested in exploring the correlational patterns between parental and youth psychopathology, especially considering parental internalizing symptoms (somatization/anxiety/depression) and offspring PTSD. Secondly, we investigated the relative contribution of parental internalizing psychopathology, the severity of exposure to earthquake, and prior trauma in affecting youth PTSD scores. Interactional effects between these variables were also explored. Finally, the relative contribution of offspring PTSD, earthquake exposure, and past trauma on parental internalizing psychopathology was analyzed. As for the previous hypothesis, interactional effects were examined.

Based on the scientific literature, an association between parental internalizing psychopathology and offspring PTSD was hypothesized. Specifically, we expected a significant contribution of parental psychopathology, the severity of exposure, and prior trauma in explaining PTSD variance in youths. Moreover, we expected offspring PTSD to have a role in parental psychopathology. Regarding the interactional analysis, no a priori hypothesis was generated due to the lack of previous studies of this kind on natural disasters.

## 2. Materials and Methods

### 2.1. Participants

Sampling included youths living in the province of Modena hit by the 2012 earthquake, and recruitment was conducted in primary and secondary schools randomly selected from the comprehensive School Regional Office register, published by the Italian Ministry of Education. To recruit participants, the province of Modena was divided into two areas: an “earthquake area” (EA), including the epicenter and the most affected zones, and a “control area” (CA), characterized by no human losses or damages to buildings due to the earthquake.

The sample of the present study, therefore, included 518 children and adolescents living in the most affected area, with a mean age of 11.21 ± 1.51 years (range 8–15 years; 48.9% males), and 325 children (mean age 11.26 ± 1.29; 52.6% males) living in the control area. With respect to sex, age, or nationality, no significant differences between the two areas were found.

A total of 1162 parents also agreed to provide information on their mental health. Parental psychopathology was examined for 474 children and adolescents in the EA (age range 29–65 ys, mean age = 43.17, SD = 5.43) and for 308 in the CA (age range 28–61 ys, mean age = 43.49, SD = 6.44). None of the youths had been previously in contact with mental health services.

### 2.2. Measures

Children and adolescents were administered an assessment protocol including:

An exposure questionnaire created ad hoc to collect demographic data, information on the degree of exposure to the earthquake (occurrence of personal, familiar, or friend injuries or death, house damages or displacement), exposure to past traumatic events (e.g., parental divorce, personal or relatives’ accidents, previous illnesses, or losses). Each item had a yes/no response format, and two indexes of exposure (earthquake exposure and lifetime traumatic exposure) were calculated. These were used as continuous in correlation analyses, while they have been dichotomized in the GLM. In the last case, a score of “1” was attributed to each youth with the severity of exposure to earthquake or a lifetime exposure over 50° percentile of the observed distribution.

The UCLA Post-traumatic Stress Disorder Reaction Index *(PTSD-RI)* for DSM-IV [77]: the most widely used instrument to assess trauma exposure and PTSD in children and adolescents exposed to any type of trauma. The 20-item version has a 5-point Likert response scale, and a total severity score can be calculated. Cronbach’s alpha was 0.90 for internal consistency across versions, and test–retest reliability was 0.84 [77]. According to the official scoring system, a cutoff > 38 was used to identify probable PTSD cases. For the purposes of this study, we derived a continuous score that was used in the analyses.

Although the Italian version of this questionnaire has not been validated, the translation process followed published guidelines, including a back-translation independently conducted by two translators.

Parents were administered the following instrument:

Symptom Checklist-90-R (SCL-90-R) [78,79], Italian version [80]. This 90-item self-report measure using a 5-point Likert scale is aimed at assessing a broad range of psychopathological disorders (according to the DSM-IV-TR). Primary dimensions include somatization, obsessive-compulsive disorder, interpersonal sensitivity, depression, anxiety, hostility, phobic anxiety, paranoid ideation, and psychoticism. For the scope of the present research, analyses focused on internalizing scales (somatization, depression, anxiety). The sum of each domain score provides a Global Severity Index, whose clinical cutoff is 63. In the Italian version, internal coherence was good for all subscales ranging between 0.70 and 0.96 [81].

### 2.3. Procedure

The study was conducted according to the guidelines of the Declaration of Helsinki and approved by the Ethical Committee of the Province of Modena (protocol n. 268/12). Two years after the earthquake, trained child psychiatrists and psychologists conducted the assessment following the authorization of the school governance (principal and board). Only children whose parents signed the informed consent and who provided their personal consent were enrolled. Subjects with likely PTSD were referred to the local child neuropsychiatric services.

### 2.4. Statistical Analysis

Means, standard deviations, frequencies, and percentages were used for descriptive data analysis. Pearson’s correlation coefficients were implemented to examine the relationship between PTSD scores, number of earthquake-related stressful events, number of prior traumatic events, and parental psychopathology scores. Finally, general linear model (GLM) was applied to explore the relative contribution of parental psychopathology, earthquake exposure, and past trauma to predict child PTSD scores. Due to the high level of collinearity among the three SCL-90-R subscales (somatization, depression, anxiety), we implemented separate models. Firstly, interaction and simple slope analyses were implemented to evaluate the interactive effects between parental psychopathology (somatization/anxiety/depression) and the variables concerning earthquake exposure and past trauma. Then, GLM was used to analyze the interactive effects between offspring PTSD scores and, respectively, the severity of exposure and past traumatic events to predict parental psychopathology (somatization/anxiety/depression). All the variables were z-transformed, and analyses were adjusted for child age and sex. Statistical analyses were performed using IBM SPSS Statistics package version 24. For all statistical tests, a *p* < 0.05 was considered statistically significant.

## 3. Results

### 3.1. Descriptive Analyses

The prevalence of probable PTSD in children and adolescents living in the area most affected by the earthquake was 1.9% (4.4% near the epicenter), and it was significantly higher than in the control zone (0.4%; *p* < 0.001). In the EA, the mean total PTSD score was also significantly higher than in the CA (15.62 ± 9.52 vs. 11.08 ± 7.50; *p* < 0.001).

While lifetime stressful events showed similar frequencies in the two areas (EA = 33.7% and CA = 37.8%, *p* = 0.16), rates (88.4%) of earthquake-related traumatic experiences in youths living in the EA were significantly higher than in the CA (19.5%; *p* < 0.001): 73.7% of children and adolescents in the EA had to leave the house for a period due to extensive damages, more than 30% had relatives or close friends who were injured or killed by the earthquake, and 4.2% experienced personal physical injuries. Moreover, about half of the youths in the EA experienced two or more events.

Nearly 1/3 of parents had high levels of psychopathology, with 28% in the EA having a global score at the SCL-90-R over the cutoff. This prevalence was higher than in the control sample (17.5%; *p* < 0.001). Significant differences were also observed in the subscales of interest for the present study: depression (22% vs. 11%; *p* < 0.001), somatization (20% vs. 16.2%; *p* = 0.0049), and anxiety (18.2% vs. 7.7%; *p* < 0.001) [82]. Mean values of all these variables are shown in Table 1, while correlations are shown in Table 2.

While in the most affected area PTSD in offspring was significantly related to their severity of exposure, past trauma, and parental psychopathology scores, in the low-affected control area PTSD did not correlate with parental internalizing psychopathology. Moreover, in both samples, parental psychopathology did not correlate with earthquake exposure and past trauma in their offspring.

### 3.2. Multivariate Analyses

In the earthquake area, GLM results (see Table 3) using youth PTSD-RI as the outcome variable revealed that parental somatization, depression, and anxiety together with earthquake exposure and past traumatic events are relevant risk factors for PTSD in children and adolescents.

In addition, we found a significant negative interaction between severity of exposure to the earthquake and parental depression or anxiety in predicting offspring PTSD symptoms. This interaction was not significant for somatization.

In the second set of models implemented with parental psychopathology as outcome (see Table 4), results showed that this was predicted by PTSD, and not by earthquake exposure nor past traumatic events experienced by their offspring. However, a negative interaction was observed between earthquake exposure and offspring PTSD in predicting depression and anxiety in parents.

The same analyses were conducted for children and adolescents living in the less affected control area (data not shown), and the severity of exposure to the earthquake resulted in the only significant predictor in all the models. No significant interactional effects were found.

In both areas, age and sex did not reach any statistical significance.

## 4. Discussion

The main aim of the present research was to explore the association between parental internalizing psychopathology and youth PTSD scores two years after the 2012 earthquake in Italy. Two subgroups of children and adolescents were compared: the first living in the most affected area and the other in a nearby control area with no human losses and damages to buildings.

As expected, correlations were found among PTSD total score in offspring and parental psychopathology, the severity of exposure, and previous traumatic events. These correlations were significant in the high-impact area but did not hold for children and adolescents living in the low-impact zone. Therefore, living in areas where losses and damages to buildings are extensive appears highly discriminant with respect to the long-term psychopathological impact of an earthquake.

These results were supported by multivariate analyses focusing on the relative contribution of parental internalizing psychopathology, the severity of exposure, and past trauma in predicting offspring PTSD. All these factors, in fact, explained a significant portion of the variance in PTSD total score, but only in the most affected area. In the other area, the only significant predictor was the degree of exposure to the earthquake. It is therefore confirmed, in accordance with recent systematic reviews and meta-analyses [15,28,29], the important role of previous traumatic events and severity of exposure to the earthquake in the development of children’s and adolescents’ PTSD. Our data collected two years after the earthquake are also in line with other studies evidencing that disaster exposure highly contributes to youth PTSD [7] up to three years later [38,60].

Evidence collected adds to a growing literature documenting the relationship between parental psychiatric difficulties and their offspring PTSD after earthquakes [41,60,61,62,63,64]. As highlighted in previous studies, parents struggling with their own psychopathology may have a limited ability to provide children and adolescents with adequate support in coping efforts and to construct a meaning for the trauma [36,47,64,66], as well as an adequate parenting style [38,67,83,84,85]. It can be therefore assumed that, for youth living close to the epicenter, exposed to human losses, personal injuries, and relocations, the impact of an earthquake is not only direct but also strengthened by the effects of other family members’ psychopathology. Although it is not possible to draw any conclusions about the mechanisms underlying the association between parental psychopathology and offspring PTSD, the absence of a significant relationship in the low-impact areas seems to suggest the important role played by environmental factors in addition to a genetic liability.

Notably, the focus on parental internalizing symptoms in the present study extends previous literature on natural disasters, mostly limited to the investigation of parental PTSD [41,61,64] or depression [62,63]. To our knowledge, none of the previous studies investigated the relationship between parental somatization and youth PTSD after natural disasters, except for one study focusing on depression in children [86].

GLM analyses highlighted a negative interactional effect between the severity of earthquake exposure and parental anxiety or depression in explaining offspring PTSD. This result suggests that the relationships among predictors of youth PTSD could be complex, not limited to the impact of single variables per se or to an additional effect. While a boosted effect between these two factors could have been hypothesized, the result of interaction analyses seems to suggest a weakening effect of one variable on the other. It might be, for example, that in case of high exposure to the earthquake, parental anxiety or depression may have a minor role in explaining PTSD variance. Vice-versa, in the presence of severe anxiety or depression in parents, the severity of exposure might provide a lower contribution in explaining variance in offspring’ psychological difficulties.

Furthermore, it is of clinical interest that the severity of exposure does not interact with parental somatization in affecting youth PTSD and that two years after the earthquake, these two variables keep an independent role. As no previous studies focused on the specific role of parental somatization on offspring PTSD, we can only suggest that, while parental anxiety and depression may interact with the severity of youth exposure to traumatic events through different cognitive processes [87], the mechanisms might be different for somatization. Future studies are needed to further explore and clarify this issue.

The third aim of the present study is found to support the hypothesis that parental psychopathology scores are predicted by offspring PTSD scores. This result is coherent with recent cross-sectional and longitudinal studies [64,72], suggesting the existence of a bidirectional pattern of influence between parental and youth psychopathology.

According to these results, not only does youth mental health rely on parents’ functioning, but also PTSD in offspring may represent a maintaining variable for parental psychopathology. As highlighted by previous studies, psychopathology in children and adolescents can be extremely stressful for parents [88,89,90]. A meta-analysis including 32 studies, for example, found a significant association between child PTSS and both parent PTSS and depression [39]. The present study suggests the need to extend these findings to parental anxiety and somatization.

Finally, GLM analyses evidenced that even though parental psychopathology is not independently predicted by youth severity of exposure and lifetime traumatic events, an interaction effect can be observed between earthquake exposure and offspring PTSD on internalizing disorders in parents: even in this model, the interactional effect was not significant for parental somatization. In the case of low or high levels of traumatic events, therefore, the contribution of offspring PTSD in explaining parental psychopathology might be different. It could be hypothesized, for example, that when a youth is severely exposed to an earthquake, his/her PTSD gives a lower contribution in explaining variance in parental depression and anxiety. This result seems in line with studies showing that, after children’s exposure to traumatic events, parents may feel guilt, shame, and a sense of hopelessness [91]. When parents feel that they failed “as a protective shield” [36], not being able to keep their child away from exposure to harm, they may develop negative emotions and concerns [87]. This “sense of failure” could manifest even in the presence of low levels of PTSD in offspring. The nature of the present study and the current state of knowledge invites cautious interpretations. The complexity of interactions among variables in traumatic situations deserves continued investigation and thoughtful study.

### Strengths, Limitations, and Future Research

This study has several strengths and limitations. It is one of the few studies carried out in Italy after an earthquake, with a large sample of children, adolescents, and parents. Furthermore, it extends findings of previous studies considering internalizing difficulties (anxiety, depression, and somatization) and having a comparison sample living in a less affected area.

In addition, both children and parents self-assessed their symptoms. As highlighted in previous studies [64,70], one of the most common methodological flaws in research assessing the effects of disasters on children’s mental health is represented by relying on parents as the only informant (with parental problems influencing the ratings of their children’s problems).

Among the methodological shortcomings, the first and most important is represented by the cross-sectional design of the study, which limits conclusions about the directionality of the effects. Another is the use of short and self-report instruments, albeit extensively used in the aftermath of natural disasters. A clinical interview would have been more appropriate than a self-report instrument in assessing offspring PTSD. Finally, trauma exposure severity and past trauma were only reported by children and adolescents (and they did not correlate with adults’ psychopathology): a more detailed assessment of parental exposure to earthquake, as well as pre-disaster data on parental and offspring psychopathology, would have allowed a better interpretation of the results.

New lines of research are needed to improve knowledge about the interactions among different variables as well as among different subjects exposed to disasters. Most importantly, longitudinal studies are needed, as the process of influence between parents and their offspring psychopathology is likely to be dynamic and not unidirectional [61]. Unfolding such trajectories will be a challenge for research in years to come.

## 5. Conclusions

Overall, the findings of the present study suggest that part of the youths exposed to natural disasters is in especially high need as not only the child but also parents are affected. Therefore, it is of primary importance to screen parents on psychopathology, providing a broader clinical assessment. As the development of PTSD in offspring appears to be significantly influenced by parental depression, anxiety and somatization, post-disaster psychological screenings should include a range of internalizing problems.

Moreover, early interventions should be offered to prevent psychopathological outcomes, giving priority to subjects living in areas most affected by earthquakes. Parents and youths should be supported as a family, not only in the aftermath of a disaster but also in the long run, and should receive treatment programs to regain hope, challenging negative cognitions and feelings that might prevent them from adaptively processing their trauma [92].

These results should be carefully considered when developing mental health interventions for children and families in areas affected by earthquakes.

## Figures and Tables

**Table 1:** Descriptive analyses of the main study variables.

	Earthquake Area M (SD)	Control Area M (SD)	*t-*Test	*p*
Offspring PTSD (CPTSD-RI)	15.62 (±9.52)	11.08 (±7.50)	−6.891	<0.001 ***
Parental somatization (SCL-90-R)	0.57 (±0.58)	0.52 (±0.47)	−1.341	0.180
Parental depression (SCL-90-R)	0.57 (±0.55)	0.45 (±0.45)	−3.371	=0.001 ***
Parental anxiety (SCL-90-R)	0.53 (±0.54)	0.40 (±0.38)	−3.923	<0.001 ***
Offspring severity of exposure to earthquake	2.24 (±0.1.17)	0.26 (±0.61)	−25.998	<0.001 ***
Offspring past traumatic events	0.52 (±0.90)	0.61 (±0.91)	1.232	0.218

Note. Significance levels *** *p* < 0.001; PTSD-RI = Post-Traumatic Stress Disorder—Reaction Index; SCL-90-R = Symptom Checklist-90-R.

**Table 2:** Correlations among the study variables in the EA and CA.

	2	3	4	5	6
1. Offspring PTSD (EA) Offspring PTSD (CA)	0.281 ** + 0.068	0.274 ** + −0.008	0.283 ** + 0.061	0.272 ** 0.223 ***	0.210 ** 0.126 *
2. Parental somatization (EA) Parental somatization (CA)	- -	0.681 ** 0.649 ***	0.731 ** 0.698 ***	0.102 0.055	0.043 0.025
3. Parental depression (EA) Parental depression (CA)		- -	0.838 ** + 0.753 ***	0.095 0.079	0.019 0.041
4. Parental anxiety (EA) Parental anxiety (CA)			-	0.111 0.117	0.025 0.052
5. Offspring severity of exposure to earthquake (EA) Offspring severity of exposure to earthquake (CA)				-	0.091 0.146 *
6. Offspring lifetime traumatic events (EA) Offspring lifetime traumatic events (CA)					-

Note. Significance levels * *p* < 0.05, ** *p* < 0.01, *** *p* < 0.001. + indicates statistically significant difference between EA and CA correlations.

**Table 3:** General linear model for the PTSD-RI in the EA.

Dependent Variable: Offspring PTSD						
ℛ² (adjusted) = 0.145						
Parameter	B	Std. Error	T	*p*	Lower Bound	Upper Bound
Offspring past traumatic events	0.416	0.109	3.821	0.000	0.201	0.603
Offspring severity of exposure to earthquake	0.297	0.105	2.818	0.005	0.089	0.504
Parental somatization	0.306	0.075	4.073	0.000	0.158	0.454
Offspring severity of exposure to earthquake * Parental somatization	−0.079	0.114	−0.691	0.490	−0.304	0.146
Offspring past traumatic events * Parental somatization	−0.060	0.112	−0.535	0.593	−0.281	0.161
Offspring sex	−0.035	0.102	−0.339	0.735	−0.236	0.167
Offspring age	0.010	0.034	0.295	0.768	−0.057	0.077
**Dependent Variable: Offspring PTSD**						
ℛ² (adjusted) = 0.141						
Parameter	B	Std. Error	T	*p*	Lower Bound	Upper Bound
Offspring past traumatic events	0.426	0.106	4.001	0.000	0.216	0.636
Offspring severity of exposure to earthquake	0.350	0.103	3.384	0.001	0.146	0.554
Parental anxiety	0.317	0.076	4.150	0.000	0.167	0.468
Offspring severity of exposure to earthquake * Parental anxiety	−0.245	0.107	−2.2923	0.023	−0.455	−0.035
Offspring past traumatic events * Parental anxiety	−0.031	0.105	−0.300	0.765	−0.238	0.175
Offspring sex	−0.047	0.100	−0.469	0.639	−0.245	0.151
Offspring age	0.010	0.033	0.321	0.749	−0.054	0.075
**Dependent Variable: Offspring PTSD**						
ℛ² (adjusted) = 0.159						
Parameter	B	Std. Error	T	*p*	Lower Bound	Upper Bound
Offspring past traumatic events	0.432	0.109	3.964	0.000	0.217	0.646
Offspring severity of exposure to earthquake	0.351	0.105	3.343	0.001	0.144	0.558
Parental depression	0.381	0.080	4.758	0.000	0.224	0.539
Offspring severity of exposure to earthquake * Parental depression	−0.313	0.111	−2.827	0.005	−0.531	−0.095
Offspring past traumatic events * Parental depression	−0.063	0.107	−0.588	0.557	−0.273	0.147
Offspring sex	−0.070	0.102	−0.684	0.495	−0.271	0.131
Offspring age	0.017	0.033	0.499	0.618	−0.049	0.083

Note: * indicates interactions between variables.

**Table 4:** General linear model for the SCL-90-R in the EA.

Dependent Variable: Parental Somatization (SCL-90-R)						
ℛ² (adjusted) = 0.086						
Parameter	B	Std. Error	T	*p*	Lower Bound	Upper Bound
Offspring past traumatic events	0.051	0.119	0.428	0.669	−0.184	0.286
Offspring severity of exposure to earthquake	0.056	0.114	0.490	0.624	−0.169	0.281
PTSD-RI	0.416	0.094	4.451	0.000	0.232	0.600
Offspring severity of exposure to earthquake * Youth PTSD	−0.174	0.123	−1.410	0.160	−0.417	0.069
Offspring past traumatic events * Offspring PTSD	−0.101	0.125	−0.809	0.419	−0.348	0.145
Offspring sex	0.066	0.110	0.599	0.550	−0.151	0.282
Offspring age	0.064	0.036	1.789	0.075	−0.006	0.135
**Dependent Variable: Parental Anxiety (SCL-90-R)**						
ℛ² (adjusted) = 0.064						
Parameter	B	Std. Error	T	*p*	Lower Bound	Upper Bound
Offspring past traumatic events	−0.013	0.122	−0.110	0.913	−0.253	0.226
Offspring severity of exposure to earthquake	−0.039	0.118	−0.330	0.742	−0.271	0.193
Offspring PTSD	0.400	0.098	4.089	0.000	0.207	0.593
Offspring severity of exposure to earthquake * PTSD-RI	−0.304	0.128	−2.377	0.018	−0.555	−0.052
Offspring past traumatic events * PTSD-RI	0.005	0.129	0.039	0.969	−0.248	0.258
Offspring sex	0.092	0.112	0.822	0.412	−0.129	0.314
Offspring age	0.049	0.037	1.334	0.183	−0.023	0.121
**Dependent Variable: Parental Depression (SCL-90-R)**						
ℛ² (adjusted) = 0.096						
Parameter	B	Std. Error	T	*p*	Lower Bound	Upper Bound
Offspring past traumatic events	0.034	0.120	0.281	0.779	−0.203	0.271
Offspring severity of exposure to earthquake	−0.066	0.116	−0.568	0.570	−0.293	0.162
Offspring PTSD	0.443	0.094	4.736	0.000	0.259	0.628
Offspring severity of exposure to earthquake * Youth PTSD	−0.391	0.124	−3.149	0.002	−0.636	−0.147
Offspring past traumatic events * Offspring PTSD	0.025	0.125	0.202	0.840	−0.221	0.272
Offspring sex	0.140	0.111	1.262	0.208	−0.078	0.357
Offspring age	0.064	0.036	1.768	0.078	−0.007	0.135

Note: * indicates interactions between variables.

## Data Availability

Due to ethical concerns, supporting data cannot be made openly available.
